# The Use of Aspirin Increases the Risk of Major Adverse Cardiac and Cerebrovascular Events in Hypertensive Patients with Obstructive Sleep Apnea for the Primary Prevention of Cardiovascular Disease: A Real-World Cohort Study

**DOI:** 10.3390/jcm11237066

**Published:** 2022-11-29

**Authors:** Nanfang Li, Wen Wen, Xintian Cai, Qing Zhu, Junli Hu, Mulalibieke Heizhati, Yujuan Yuan, Lin Gan, Yujie Dang, Wenbo Yang, Jing Hong, Xiangyang Zhang

**Affiliations:** 1 Hypertension Center of People’s Hospital of Xinjiang Uygur Autonomous Region, Xinjiang Hypertension Institute, NHC Key Laboratory of Hypertension Clinical Research, Key Laboratory of Xinjiang Uygur Autonomous Region, Xinjiang Clinical Medical Research Center for Hypertension Diseases, Urumqi 830000, China; 2 School of Graduate Studies, Xinjiang Medical University, Urumqi 830000, China; 3 The First Affiliated Hospital of Xinjiang Medical University, Urumqi 830000, China

**Keywords:** obstructive sleep apnea, hypertension, aspirin, cardiovascular diseases, primary prevention, real world study

## Abstract

(1) Background: Hypertensive patients with obstructive sleep apnea (OSA) are at high risk for cardiovascular diseases (CVDs), and the utility of aspirin for primary cardiovascular prevention in this population remains uncertain. (2) Methods: In this retrospective cohort study using data from the *Urumchi Hypertension Database* (UHDATA), hypertensive patients older than 18 years old with a first-time diagnosis of OSA were divided into three groups depending on aspirin history. Major adverse cardiac and cerebrovascular events (MACCE) were the primary outcome. Secondary outcomes included MACCE components, ischemic events, cardiac events, cerebrovascular events, and gastrointestinal bleeding risk. The inverse probability of treatment weighting (IPTW) method was used to balance the confounding factors among the groups, and the Cox proportional hazards model was used to calculate the hazard ratio (HR) and 95% confidence interval (CI). (3) Results: In persistent aspirin users, the risk of MACCE events (HR 2.11, 95%CI 1.23–3.63), ischemic events (HR 2.58, 95%CI 1.42–4.69), cerebrovascular events (HR 2.55, 95%CI 1.44–4.51), and non-fatal cerebral infarction (HR 3.14, 95%CI 1.69–5.84) was significantly elevated. (4) Conclusions: Continuous aspirin use increases the incidence of cardiovascular adverse events in hypertensive patients with OSA receiving aspirin for primary prevention of cardiovascular disease.

## 1. Introduction

Cardiovascular diseases (CVDs) have a high prevalence and mortality rate globally and in China, posing a significant economic burden and health risk to society [1,2]. Patients with hypertension and obstructive sleep apnea (OSA) are both high-risk groups for CVDs [3,4]. At the same time, the comorbidity rate of hypertension and OSA is very high, and the risk of cardiovascular adverse events in patients with hypertension combined with OSA is significantly higher than in patients with isolated hypertension or OSA alone [5]. Therefore, in addition to secondary prevention of cardiovascular disease, primary prevention for high-risk groups is an effective strategy for reducing the incidence of CVDs [2].

The role of aspirin antiplatelet aggregation therapy in the secondary prevention of CVDs is well established [6], but the role of aspirin in the primary prevention of CVDs is debatable. According to a 2016 U.S. Preventive Services Task Force (USPSTF) report, aspirin should be recommended for the primary prevention of cardiovascular disease in people at high risk of CVDs [7]. However, in the same year, European guidelines did not recommend the use of aspirin in patients without a history of CVDs [8]. The 2019 American College of Cardiology/American Heart Association (ACC/AHA) guidelines also advise against using aspirin for primary prevention in people aged < 40 and ≥70 [9]. A growing number of studies suggest that aspirin does not reduce cardiovascular mortality and may even increase the risk of bleeding or that the harms of aspirin outweigh its benefits [10,11].

Hypertensive patients with OSA have a unique pathophysiological mechanism. The release of inflammatory factors in patients with hypertension and OSA increases due to long-term chronic intermittent hypoxia and activation of the renin-angiotensin-aldosterone system (RAAS), and vascular endothelial injury is exacerbated. It further stimulates the platelet aggregation process, making hypertensive and OSA patients more susceptible to ischemic cardiovascular adverse events [12,13]. Although the cardiovascular guidelines recommend primary prevention with aspirin for high-cardiovascular-risk groups, the evaluation of the effect of aspirin and the cut-off point for initiating treatment for the special group of patients with hypertension complicated by OSA are still unclear in the relevant guidelines [14,15]. Therefore, this study used a retrospective cohort to explore the relationship between aspirin use for primary prevention and major adverse cardiovascular and cerebrovascular events (MACCE) in hypertensive patients with OSA who were at high cardiovascular risk.

## 2. Materials and Methods

### 2.1. Data Sources

*Hypertension Database in Urumchi* (UHDATA) is a specialized database for hypertension in Urumqi that was created by The Key Laboratory for Hypertension Clinical Research and The National Health Committee of China. It relies primarily on the electronic medical record system of the Xinjiang Uygur Autonomous Region People’s Hospital, which is a comprehensive electronic medical database comprised of multiple systems, such as the imaging radiology system, laboratory information system, pathological system, and ultrasound system [16]. This database includes patient personal information, all outpatient and inpatient visit information, diagnosis information, medical records, laboratory test results, prescription and medical order information, cost information, etc., and can retrieve medical records information that meets the inclusion criteria and the aforementioned criteria based on predefined fields. The database contains all patients diagnosed with hypertension at the People’s Hospital of Xinjiang Uygur Autonomous Region since January 2004. According to the 10th edition of the International Classification of Disease (ICD-10), hypertension is coded as I10-I15. The medicines and diagnostic data used during the follow-up are primarily from Urumqi’s social medical insurance system, which includes inpatient and outpatient diagnosis information, death information, drug prescribing information, and cost information. The retrieved data contain patient-specific information such as gender, age, etc., as well as information regarding inpatient consultations and pharmacy visits, such as disease diagnosis, status, prescription medicines, etc. The study protocol has been approved by the Ethics Committee of the Xinjiang Uygur Autonomous Region People’s Hospital, and the research strictly adhered to the ethical standards of the Declaration of Helsinki.

### 2.2. Study Population

**Figure 1:**
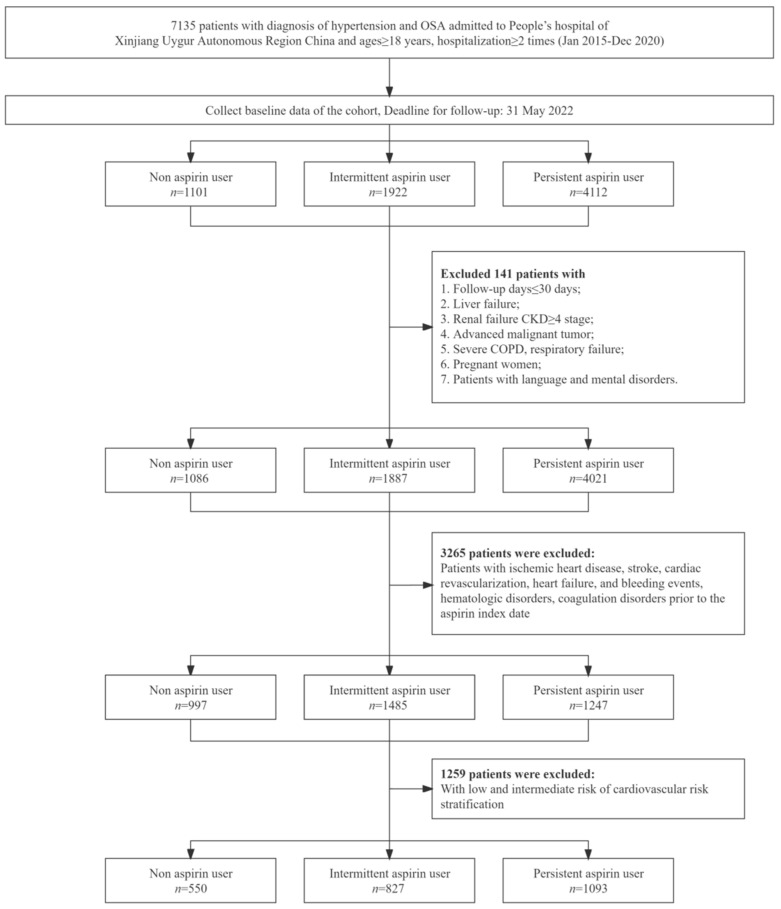
Participants Flowchart. Annotation: OSA, obstructive sleep apnea; CKD, chronic kidney disease; COPD, chronic obstructive pulmonary diseases.

The database was searched for hypertensive individuals (I10-I15) who were initially diagnosed with obstructive sleep apnea (I27.9) between 1 January 2015 and 31 December 2020, using the ICD-10 code. There were 7135 patients aged ≥ 18 with hospitalization times ≥ 2. The population was separated into three groups based on their usage of aspirin. As determined by the number of aspirin prescriptions in the social medical insurance database, the aspirin group consists of persons who have never taken aspirin throughout the study period (aspirin prescriptions in inpatient orders, outpatient clinics, and pharmacy records are all 0), intermittent aspirin users (aspirin prescription: long-term inpatient doctor’s order 1 time/year + 0 times ≤ outpatient or pharmacy prescription ≤ 2 times/year or long-term inpatient doctor’s order 0 times/year + 1 ≤ outpatient or pharmacy prescription ≤ 3 times/year), and persistent aspirin users (aspirin prescription: inpatient long-term medical orders ≥ 2 times/year + outpatient or pharmacy prescriptions ≥2 times/year, inpatient long-term medical orders 1 time/year + outpatient or pharmacy prescriptions ≥ 3 times/year, or inpatient long-term medical orders 0 times/year + pharmacy prescription ≥ 4 times/year). Excluded were 141 patients with ≤30 days of follow-up and serious underlying conditions, including liver failure, chronic kidney disease (CKD) stage > 4, advanced malignant tumor, severe chronic obstructive pulmonary disease, respiratory failure, pregnancy, and mental disorder. Individuals taking aspirin were indexed based on the date of their first aspirin prescription, whereas non-users were indexed based on the baseline date when cases were added. Patients with the following underlying medical conditions before the index date were excluded: ischemic heart disease (I20-I25), cardiac revascularization (International Classification of Diseases Clinical Modification of 9th Revision Operations and Procedures—ICD-9-CM-3 codes: 36.0-36.1, 00.45-00.48, 00.66, 17.55), heart failure (I50), stroke (I61-I63), and gastrointestinal bleeding events (K92.0-K92.2), totaling 3265 cases. A total of 2470 patients with high cardiovascular risk stratification for primary prevention of cardiovascular disease were screened among the remaining patients. Figure 1 depicts the inclusion and exclusion criteria in detail.

### 2.3. Data Collection and Definition

We acquired patient information from UHDATA as of 31 May 2022. General data (age, gender, smoking, and drinking), anthropometric data (body mass index, systolic, diastolic, and waist circumference), biochemical measurements (total cholesterol, triglycerides, high-density lipoprotein cholesterol, and low-density lipoprotein cholesterol), serum creatinine (Crea), blood urea nitrogen (BUN), blood uric acid (UA), alanine aminotransferase (ALT), aspartate aminotransferase (AST), clinical data (duration of hypertension, OSA classification, cardiovascular risk stratification), comorbidities (coronary heart disease, heart failure, cerebrovascular illness, diabetes, chronic kidney disease, unspecified kidney failure, and gastrointestinal bleeding, according to ICD-10 codes I20-I25, I50, I60-I66, I69, E10-E11, N18, N19, K92.0-K92.2), cardiac revascularization operations (ICD-9-CM-3: 36.0-36.1, 00.45-00.48, 00.66, 17.55), and history of drug uses (including angiotensin converting enzyme inhibitors (ACEI), angiotensin receptor blockers (ARB), beta-receptor blockers (β-receptor blocker), calcium channel blockers (CCBs), diuretics, antidiabetic, hypolipidemic, and antithrombotic drugs). The apnea hypopnea index (AHI) was defined as the sum of apneas and hypopneas per hour of sleep. The severity of OSA was classified as mild (5 ≤ AHI < 15), moderate (15 ≤ AHI < 30), and severe (AHI ≥ 30) based on clinical diagnosis. The 2010 edition of the Chinese Guidelines for the Prevention and Treatment of Hypertension defines cardiovascular risk stratification for hypertension [17]. According to habit, smoking and drinking were characterized as never and ever.

### 2.4. Follow-Up and Results

All patients’ outpatient or inpatient medical records were mined for follow-up information. The collection of follow-up data concluded on 31 May 2022. Patients in this trial were followed for a maximum of 7.4 years from the index date until the occurrence of the study outcome, death, or the end of follow-up (whichever occurred first). MACCE included all-cause death, non-fatal myocardial infarction (I21-I22), non-fatal hemorrhagic and ischemic stroke (I61-I63), and cardiac revascularization (ICD-9-CM-3 codes: 36.0-36.1, 00.45-00.48, 00.66, 17.55) as the primary endpoint. Components of MACCE, ischemic events (defined as the total of nonfatal myocardial infarction, cardiac revascularization, and ischemic stroke), cardiac events (defined as cardiac death, nonfatal myocardial infarction, and cardiac revascularization), and cerebrovascular events (defined as the sum of cerebrovascular death and hemorrhagic and ischemic stroke) were secondary outcomes. All results were derived from hospital diagnostic data. Results for end-point occurrences that happened within 30 days of the index date were not recorded to evaluate if they were connected to aspirin consumption. We evaluated the risk of hemorrhagic stroke (I60-I62) and gastrointestinal bleeding to determine the drug’s safety (K92.0-K92.2). Additionally, hemorrhagic stroke and inpatient gastrointestinal bleeding were considered as hospitalization-required bleeding episodes.

### 2.5. Statistical Analysis

In observational research, missing data are unavoidable. In this dataset, missing data accounted for 6.76% of all covariates (Supplementary Table S1). To reduce the bias caused by missing data, this study applied multiple imputation method to fill in the missing data and compared the interpolated data with the original data [18,19].

The Kolmogorov–Smirnov test was applied to test the normality of the data. Continuous variables were reported as mean standard deviation (SD) or median and interquartile range (IQR), and the ANOVA and Kruskal–Wallis test were utilized to compare groups. Categorical variables were expressed as observations and percentages, and Pearson’s chi-square test was used to compare groups. The cumulative incidence of the primary outcome was computed using Kaplan–Meier curves, and the log-rank test was utilized to determine group differences. Multivariate Cox proportional hazards models were used to find independent predictors of endpoint events, and their components were adjusted for known endpoint event-influencing factors. We balanced the confounders between groups using inverse probability of treatment weighting (IPTW) and reproduced the results using a Cox regression model weighted with the same weights (inverse probability-weighted Cox) [18,19]. Stratified analysis was conducted according to gender, age, body mass index (BMI), smoking, drinking, UA, duration of hypertension, OSA grade, baseline diabetes, and drug types. E-values were calculated to measure the effect of unmeasured confounding variables on the results [20,21]. Data were analyzed using R (version 4.2.1; R Foundation for Statistical Computing, Vienna, Austria) statistical software, all analyses were two-tailed, and *p* < 0.05 was considered statistically significant.

## 3. Result

### 3.1. Baseline Characteristics of the Participants

According to the number of aspirin prescriptions, the participants were categorized as non-users, intermittent users, and continuous users. Before and after IPTW, Table 1 describes the participants’ baseline characteristics. Age, duration of hypertension, baseline diastolic blood pressure (DBP), BMI, waist, baseline diabetes, high-density lipoprotein cholesterol, hypoglycemic drugs, lipid-lowering drugs, ACEI/ARB, β-receptor blocker, CCBs, and diuretics demonstrated significant differences between groups before IPTW. Gender, smoking, drinking, baseline systolic blood pressure (SBP), OSA grade, ALT, AST, Crea, BUN, UA, TC, TG, and LDLC were not significantly different between the three groups. The persistent aspirin users were older (50.6 ± 8.9 years), had a longer duration of hypertension (6.1 ± 3.1 years), had lower diastolic blood pressure (91.3 ± 15.0 mmHg), and had a higher prevalence of diabetes at baseline among the three groups (29.4% vs. 23.6% vs. 22.2%). Additionally, the proportion of individuals taking multiple medications is higher in the persistent aspirin users. After IPTW, there were no statistically significant differences between the groups.

### 3.2. Relationship between Aspirin and Endpoint Events

Overall, 2470 patients were followed for an average of 42.3 months, and 246 incidents of MACCE occurred, including 6 cases of all-cause death, 13 cases of non-fatal myocardial infarction, 32 cases of revascularization, 14 cases of non-fatal cerebral hemorrhage, and 203 cases of non-fatal cerebral infarction.

**Figure 2:**
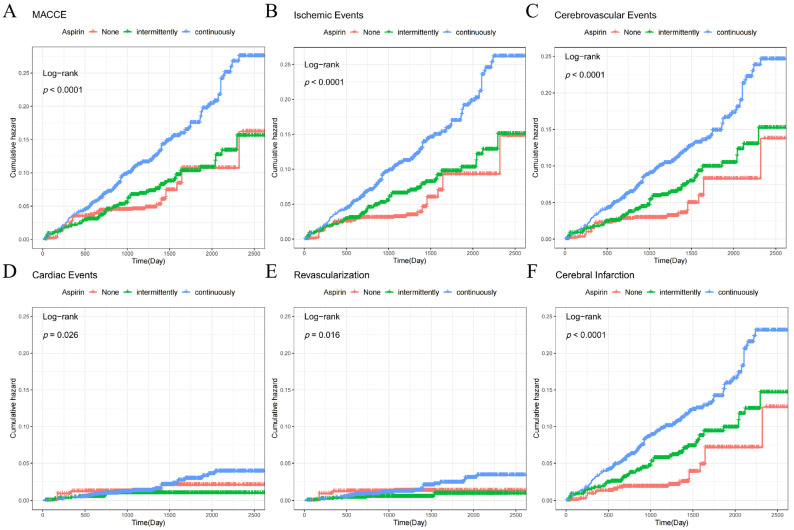
Cumulative incidence of aspirin groups and each end-point event assessed by Kaplan–Meier curves. Annotation: (**A**) MACCE; (**B**) ischemic Events; (**C**) cerebrovascular events; (**D**) cardiac events; (**E**) revascularization; (**F**) cerebral infarction. MACCE, major adverse cardiovascular and cerebrovascular events.

According to aspirin groups, modified Kaplan–Meier curves for the cumulative incidence of events were produced using IPTW (Figure 2). The cumulative incidence of MACCE events, ischemic events, cerebrovascular events, cardiac events, revascularization, and non-fatal ischemic cerebral infarction was shown to be greater (*p* < 0.05) in the persistent aspirin users’ group. In terms of all-cause death, non-fatal myocardial infarction, and non-fatal hemorrhagic stroke, there was no statistical difference between the three groups (*p* > 0.05), and there was no statistically significant difference between the non-users and persistent users in terms of gastrointestinal bleeding events (Figure S1). Based on univariate Cox, we selected factors with *p* < 0.1 and included multivariate Cox models for analysis. In model 1, we adjusted for age, gender, smoking, baseline systolic blood pressure, and BMI; in model 2, we further adjusted for medication use based on model 1. To adjust for confounding factors, a full-model Cox analysis was conducted based on IPTW. The persistent aspirin users’ group had a significantly higher risk of MACCE events (HR 2.11, 95%CI 1.23–3.63), ischemic events (HR 2.58, 95%CI 1.42–4.69), cerebrovascular events (HR 2.55, 95%CI 1.44–4.51), and non-fatal cerebral infarction (HR 3.14, 95%CI 1.69–5.84). These findings were not found in the intermittent aspirin users’ group. There were no statistically significant differences between the intermittently and persistently aspirin users’ groups in terms of all-cause death, cardiac events, nonfatal myocardial infarction, cardiac revascularization, cerebral hemorrhage, or gastrointestinal bleeding (Table 2).

### 3.3. The Relationship between Aspirin and Hemorrhagic Stroke and Gastrointestinal Bleeding

To clarify the risk of bleeding associated with aspirin use, we additionally monitored patients with hemorrhagic stroke and gastrointestinal bleeding. During the follow-up period, there were 14 hemorrhagic strokes and 33 gastrointestinal bleeding incidents. According to multivariate Cox analysis after IPTW, the results indicated that aspirin medication is not associated with increased risk of cerebral hemorrhage (HR 0.48, 95%CI 0.10–2.29; HR 0.33, 95%CI 0.07–1.51) and gastrointestinal hemorrhage (HR 0.31, 95%CI 0.08–1.16; HR 0.66, 95%CI 0.23–1.86) in either the intermittent or persistent users’ groups (Table 2).

### 3.4. Relationship between Aspirin Use and Endpoint Events in Patients with Different Grades of OSA

We investigated the relationship between aspirin use and each endpoint event in patients with mild, moderate, and severe OSA. The results suggest that in patients with mild OSA, continuous aspirin use increases the risk of ischemic events (HR 6.42, 95%CI 2.22–18.61), cerebrovascular events (HR 7.61, 95%CI 2.40–24.10), and cerebral infarction (HR 8.76, 95%CI 2.47–31.10; *p* for interaction = 0.026, 0.047, and 0.033, respectively). This risk was not significant in patients with moderate and severe OSA. In addition to this, the risk of MACCE, ischemic events, cerebrovascular events, cerebral infarction, and gastrointestinal bleeding in patients taking intermittent aspirin was not significantly different in the mild, moderate and severe OSA subgroups (*p* for interaction > 0.05, Supplementary Table S2). We further combined patients with moderate and severe OSA and obtained a trend similar to the above results (Supplementary Table S3). Not only that, but when we compared patients with moderate and severe OSA separately, we found no significant effect of OSA classification on the risk of endpoint events when either intermittent or continuous aspirin was administered (*p* for interaction > 0.05, Supplementary Table S4). The relationship between aspirin use and all-cause death, cerebral hemorrhage, revascularization, myocardial infarction, and cardiac events was not further explored in patients with different grades of OSA because of the influence of statistical efficacy.

### 3.5. Sensitivity and Stratified Analysis

We utilized multiple imputation to account for missing data and then compared baseline data before and after multiple imputation. There was no significant difference between the results before and after multiple imputation, as shown by *p* > 0.05 in Supplementary Table S5. Analysis was conducted according to the following predetermined subgroups: gender (male, female), age (<50 years, ≥50 years), BMI (<28 kg/m^2^, ≥28 kg/m^2^), smoke (never, ever), drink (never, ever), UA (≤420 μmol/L, >420 μmol/L), duration of hypertension (≤4 years, 4–8 years, and >8 years), baseline diabetes (yes, no), and medication status. In the group of persistent aspirin users, patients aged ≥ 50 had a significantly higher risk of MACCE events (*p* for interaction = 0.001, Supplemental Table S6). Using ischemic events as the endpoint, we repeated the subgroup analysis described before. The incidence of ischemia events was significantly elevated among patients aged 50 years who continued to take aspirin (HR 3.39, 95%CI 1.58–7.28, *p* for interaction = 0.003, Supplemental Table S7). After age stratification, the association between aspirin use and each endpoint was assessed. The results indicated that patients aged ≥ 50 years will have an increased risk of MACCE, ischemic events, cerebrovascular events, cerebral infarction, and cardiac events (*p* for interaction = 0.001, 0.003, 0.012, 0.031, and 0.029, respectively). In persistent aspirin users, < 50 years old also had an increased risk of cerebrovascular events (HR 2.49, 95%CI 1.15–5.41) and cerebral infarction (HR 2.77, 95%CI 1.19–6.42). After using aspirin, the risk of cerebral hemorrhage and gastrointestinal bleeding did not change with age (Supplementary Table S8). Calculation of E-values for study endpoints and comparison with HR estimates for statistically significant endpoint events after IPTW indicated that unmeasured confounders are unlikely to account for the observed association between persistent aspirin use and each endpoint (Supplementary Figure S2 and Table S9). Due to insufficient statistical power, additional subgroup analyses were not conducted for other endpoints.

## 4. Discussion

Hypertensive patients with OSA are a high-risk category for CVDs. However, the use of aspirin for primary prevention in this population is currently unknown. The 2022 USPSTF Recommendations in the Primary Prevention of Cardiovascular Disease Population state that the net benefit of aspirin use is small in people aged 40 to 59 with a cardiovascular disease risk of > 10%, and it is advised that persons ≥ 60 years old not begin low-dose aspirin for primary prevention of CVDs [22]. We investigated the association between aspirin use history and MACCE in patients with hypertension and OSA for the primary prevention of CVDs. Our investigation found that in hypertensive patients with OSA for primary prevention, prolonged aspirin usage was related to an increased risk of MACCE events and that this risk was greater in individuals ≥50 years of age and in patients with mild OSA.

The proportion of ischemia events in our study population was higher than that of other endpoints, which was attributed to the pathophysiological mechanisms underlying OSA and hypertension. Chronic intermittent hypoxia is the fundamental pathophysiological cause of obstructive sleep apnea. In addition to initiating the production of inflammatory chemicals that might damage the vascular endothelium, OSA can also significantly amplify platelet autoreactivity, which is one of the reasons why OSA patients are susceptible to thrombosis [23]. After detecting blood coagulation factors such as von Willebrand factor (VWF), fibrinogen, and D-dimer in the plasma of OSA patients, Von et al. discovered that the coagulation factor of OSA patients was significantly higher than that of non-OSA patients [24]. The hypercoagulable status of OSA patients may be one of the key reasons that make this population more susceptible to ischemic events. The RAAS system is activated in hypertension individuals, and the malfunction of vascular endothelial cells can exacerbate the damage of vascular endothelium and promote the process of platelet aggregation [25]. Therefore, we hypothesize that “increased platelet aggregation”, “increased platelet reactivity”, and “blood hypercoagulability” are significant contributors to the occurrence of severe ischemic events in individuals with hypertension and OSA. The researchers also discovered that using aspirin did not reduce the risk of adverse cardiovascular events in individuals with hypertension and OSA but rather increased the risk of MACCE, ischemic events, cerebrovascular events, and non-fatal stroke. Similar to our work, Gong et al. discovered that dual antiplatelet therapy increases the risk of atherosclerotic thrombosis in OSA patients with acute coronary syndrome, which is directly associated with the increased platelet reactivity following antiplatelet drug treatment [26]. Jiang et al. also observed these results [27]. The results of Scinico et al. indicate that the rate of aspirin resistance in OSA patients is 17% and that the patient’s responsiveness to aspirin has improved following therapy with continuous positive airway pressure (CPAP) [28]. This shows that aspirin did not provide the predicted protection for OSA patients. In addition, due to long-term chronic hypoxia, the compensatory increase of red blood cells in patients with OSA and red blood cells will increase the platelet aggregation-promoting effect, resulting in the insufficient reduction of platelet aggregation reactivity despite taking 200–300 mg of aspirin [29]. Additionally, the hemodynamic disturbance caused by repeated apnea will enhance the oscillatory shear stress of the vessel wall, which will damage the fragile plaque and the stability of the thrombus, and the use of antiplatelet medications may cause the thrombus to break off more easily [13,30]. These are most likely the reasons why our study’s target population did not benefit from aspirin. Therefore, the use of aspirin for primary cardiovascular protection in individuals with hypertension and OSA remains controversial. In addition, the dosage of aspirin warrants further examination in light of the aforementioned research findings and perspectives because in the actual world, the majority of hypertensive patients with OSA take low-dose aspirin (75–100 mg/d) for primary CVD protection. It is common knowledge that low-dose aspirin inhibits the activity of cyclooxygenase-1 and the formation of thromboxane (TX)A_2_ in platelets, resulting in a decrease in the ratio of TXA_2_ to prostaglandin (PG)I_2_ and exerting an antithrombotic effect [31]. However, the TXA2 in the circulation of patients with hypertension and OSA is higher than that of normal people, the ratio of TXA_2_/PGI_2_ is increased [32,33], and low-dose aspirin is insufficient to block the platelet aggregation process in this patient group and even destroys the original ratio of TXA_2_/PGI_2_ in patients’ balance. Therefore, for primary prevention in this population, it merits additional investigation whether the aspirin dose should be increased or whether personalized treatment should be done to obtain advantages.

Numerous large randomized controlled trials (RCTs) on aspirin as a primary prophylaxis for CVD have demonstrated that not only does long-term aspirin use not significantly lower the risk of CVD events, but it also increases the risk of bleeding, particularly gastrointestinal bleeding [10,34,35]. In this research, the endpoints of nonfatal cerebral hemorrhage and gastrointestinal bleeding were observed in patients with hypertension and OSA. We found no increased risk of bleeding in either the intermittent or continuous aspirin use groups. This contradicts the current aspirin RCT outcomes (JPPP [34], ASPREE [10], ARRIVE [35]). This factor is related to the following considerations: First, the participants in ARRIVE and ASPREE were predominantly Caucasian and from European and American countries, which is different from the Asian ethnicity we investigated. Second, although the participants in JPPP were of Asian ethnicity, the participants in JPPP were aged 60–85 years; the participants in ASPREE were all elderly people aged ≥70 years (including a small number of Latino minorities aged ≥65 years), and the participants in ARRIVE were also older (>55 years for men and >60 years for women); in the present study, the majority of participants were between 40 and 50 years of age, and the risk of bleeding was significantly lower in this age group than in the older population. Third, in ARRIVE, participants were at moderate risk for CVD without diabetes; in ASPREE, participants were healthy community populations; and participants were those with hypertension, dyslipidemia, or diabetes in JPPP. In our study, the participants were hypertensive with OSA, a group that is prone to “hypercoagulable state”, and low-dose aspirin may not be able to counteract the hypercoagulable state of the blood so that it cannot cause bleeding. However, the median follow-up time in this trial was only 3.5 years, and we do not know if the patient’s risk of bleeding will increase after taking the treatment for a longer period; therefore, extended observation and follow-up are still required.

In addition, a stratified analysis showed that continuous aspirin treatment in patients with mild OSA significantly increased the risk of ischemic events, suggesting that physicians should evaluate patients with mild OSA carefully before prescribing aspirin. This may be attributable to the fact that CPAP therapy reduces platelet reactivity and improves aspirin resistance in patients with moderate to severe OSA [28,36,37]. However, our data lack follow-up information regarding CPAP therapy. In the next stage, we will also investigate the association between the role of CPAP therapy in antiplatelet therapy and the occurrence of MACCE episodes.

In this study, in addition to our finding that aspirin consumption increases the risk of adverse cardiovascular events, we also paid special attention to the risk stratification of patients in different age groups and found that in people aged ≥50 years, primary prevention with aspirin will not be advantageous but will be associated with increased potential risks, particularly the likelihood of ischemic events. Age is, in general, an independent risk factor for cardiovascular complications [1]. Controversy surrounds the determination of aspirin use in the primary prevention group. The 2019 ACC/AHA guidelines advocate avoiding aspirin use in the ≥70-year-old primary prevention population [9]. The USPSTF’s most recent statement lowered the upper age restriction for avoiding aspirin as a primary CVDs preventive medicine to 60 years [22]. However, unlike our investigation, the population in the above-mentioned trial did not have a co-morbid state of hypertension and OSA; therefore, the optimal age of aspirin for primary prevention of hypertensive patients with OSA remains unknown. Based on our findings, continued aspirin use significantly raised the incidence of MACCE events, ischemic events, cardiac events, cerebrovascular events, and ischemic stroke in individuals ≥50 years old. This shows that the use of aspirin for primary prevention in hypertensive patients with OSA above the age of 50 may be unwise. However, additional evidence is still required. In addition, regardless of age, the risk of cerebrovascular episodes and ischemic stroke increased in patients who continued to use aspirin. Does this imply that addressing these people with aspirin for primary cardiovascular prevention may raise their risk of adverse cardiovascular events? We recommend using caution when prescribing aspirin to hypertensive and OSA patients in primary prevention. First, clinicians should focus more on personal history (e.g., history of prematurity, first-degree family history of CVDs, presence of comorbidities, etc.) when assessing cardiovascular risk in patients with OSA in order to obtain a more individualized treatment plan. Second, patients’ CVDs risk factors should be controlled in a multidimensional manner (e.g., lifestyle improvement, removal of biological risk factors, treatment of comorbidities, etc.) rather than being limited to drug-level preventive measures. Again, the risk of CVDs is a dynamic process, and therefore, for aspirin use, it should also be dynamic; clinicians should periodically reevaluate indications and/or risk of adverse bleeding to maximize the benefit of treatment for patients during the risk dynamic process. Finally, the use of aspirin should respect the patient’s wishes and treatment expectations.

In summary, (1) different from other studies, aspirin did not increase the risk of bleeding in hypertensive patients with OSA in this cohort. (2) Nevertheless, in this cohort, aspirin did not significantly prevent MACCE. (3) Therefore, based on the presented data, aspirin cannot be recommended as a primary prevention strategy in this cohort. (4) To clarify the deep relationship between aspirin and MACCE in hypertensive patients with OSA, it is necessary to conduct a multi-dimensional exploration of aspirin dose, duration of treatment, and patient subgroups/phenotypes in a prospective manner.

Our study is limited by the following factors: First, this is a single-center observational study, which has the drawbacks of non-randomized studies. To confirm the validity of the data, we applied statistical methods of post-randomization—IPTW—to equalize confounding factors, calculated E-values to assess unmeasured confounders, and adjusted for multivariate. Second, the low endpoint event rates found in this sample were mostly a result of this population’s primary cardiovascular preventive status, milder disease, and younger age. Additionally, there was a brief follow-up period. Therefore, we will continue to follow-up with this cohort and collect more follow-up data. Third, our follow-up study lacked information on CPAP therapy and other medication therapy. This precluded us from confirming the study of additional factors that may have affected the outcome. Despite these limitations, to the best of our knowledge, this is the first large-scale cohort research investigating the link between aspirin use and MACCE events in hypertensive individuals with OSA for primary cardiovascular prevention. Furthermore, it was discovered that the continued use of aspirin may not assist patients in this category but rather increase their risk. Our findings have substantial implications for the clinical treatment of hypertensive and OSA patients in cardiovascular primary prevention groups.

## 5. Conclusions

Although our study is from the real world and has the limitations of non-randomized trials, current statistical results indicate that the use of aspirin for primary prevention in hypertensive OSA patients with a high risk of cardiovascular disease is associated with increased risks of cardiovascular adverse events. Although these are patients at high risk of cardiovascular disease, for which aspirin is recommended in European or U.S. guidelines [7,8,9,22], more attention should be paid to identifying the cause in the actual treatment evaluation, and the decision to prescribe aspirin should be based on whether the patient has OSA. We suggest caution in the use of aspirin for primary prevention in hypertensive patients with OSA. Our findings not only broaden the content of the advantages and drawbacks of primary prevention with aspirin in certain illness categories but also suggest new paths for primary prevention treatment approaches in this population.

## Figures and Tables

**Table 1:** Participants’ baseline characteristics.

	All	Non-Users	Intermittent Users	Persistent Users	*p* before IPTW	*p* after IPTW
	*n* = 2470	*n* = 550	*n* = 827	*n* = 1093		
Gender					0.829	0.528
Female	558 (22.6%)	125 (22.7%)	192 (23.2%)	241 (22.0%)		
Male	1912 (77.4%)	425 (77.3%)	635 (76.8%)	852 (78.0%)		
Age, y	48.1 (9.8)	43.7 (10.1)	47.8 (9.8)	50.6 (8.9)	<0.001	0.758
Duration of hypertension, y	5.8 (3.1)	5.3 (3.1)	5.7 (3.1)	6.1 (3.1)	<0.001	0.953
Duration of hypertension groups					<0.001	0.345
≤4 years	735 (29.8%)	200 (36.4%)	258 (31.2%)	277 (25.3%)		
>4 years, ≤8 years	994 (40.2%)	218 (39.6%)	313 (37.8%)	463 (42.4%)		
>8 years	741 (30.0%)	132 (24.0%)	256 (31.0%)	353 (32.3%)		
Smoke (ever)	1209 (48.9%)	267 (48.5%)	395 (47.8%)	547 (50.0%)	0.598	0.199
Drink (ever)	1108 (44.9%)	240 (43.6%)	360 (43.5%)	508 (46.5%)	0.353	0.966
Baseline DBP, mmHg	92.9 (15.2)	94.5 (15.8)	94.0 (14.8)	91.3 (15.0)	<0.001	0.611
Baseline SBP, mmHg	149 (21.5)	149 (21.6)	149 (21.3)	148 (21.6)	0.518	0.683
BMI, kg/m^2^	28.9 (4.5)	29.2 (5.3)	29.1 (4.7)	28.5 (3.7)	0.001	0.733
BMI groups					0.472	0.440
<28 kg/m^2^	1114 (45.1%)	242 (44.0%)	364 (44.0%)	508 (46.5%)		
≥28 kg/m^2^	1356 (54.9%)	308 (56.0%)	463 (56.0%)	585 (53.5%)		
Waist, cm	102 (11.8)	103 (13.7)	103 (12.1)	102 (10.5)	0.047	0.855
OSA grade					0.281	0.540
Mild	862 (34.9%)	204 (37.1%)	292 (35.3%)	366 (33.5%)		
Moderate	764 (30.9%)	160 (29.1%)	242 (29.3%)	362 (33.1%)		
Severe	844 (34.2%)	186 (33.8%)	293 (35.4%)	365 (33.4%)		
Baseline DM	639 (25.9%)	123 (22.4%)	195 (23.6%)	321 (29.4%)	0.002	0.826
ALT, U/L	26.0 [18.0; 39.0]	26.0 [18.7; 41.9]	26.0 [17.9; 40.0]	25.2 [18.0; 38.0]	0.228	0.777
AST, U/L	19.0 [16.0; 25.0]	19.9 [16.0; 25.0]	19.0 [15.5; 24.0]	19.0 [16.0; 25.0]	0.534	0.788
Crea, μmol/L	68.1 [58.7; 78.4]	69.5 [58.6; 78.7]	67.9 [59.0; 78.4]	67.7 [58.7; 78.0]	0.243	0.856
BUN, mmol/L	5.0 [4.2; 5.9]	5.0 [4.2; 5.9]	4.9 [4.1; 5.9]	5.0 [4.2; 5.9]	0.745	0.895
UA, μmol/L	369 [309; 431]	377 [320; 441]	370 [307; 431]	366 [307; 428]	0.062	0.973
TC, mmol/L	4.5 [3.9; 5.1]	4.4 [3.8; 5.1]	4.5 [3.9; 5.1]	4.5 [3.9; 5.2]	0.116	0.723
TG, mmol/L	1.8 [1.3; 2.6]	1.9 [1.3; 2.8]	1.8 [1.3; 2.6]	1.8 [1.7; 2.6]	0.060	0.931
HDLC, mmol/L	0.9 [0.8; 1.1]	0.9 [0.8; 1.0]	0.9 [0.8; 1.1]	0.9 [0.8; 1.1]	<0.001	0.958
LDLC, mmol/L	2.7 [2.2; 3.3]	2.6 [2.2; 3.2]	2.7 [2.2; 3.2]	2.7 [2.2; 3.3]	0.184	0.920
Hypoglycemic drugs	1461 (59.1%)	249 (45.3%)	475 (57.4%)	737 (67.4%)	<0.001	0.862
Lipid-lowering drugs	2041 (82.6%)	346 (62.9%)	648 (78.4%)	1047 (95.8%)	<0.001	0.912
ACEI/ARB	1926 (78.0%)	350 (63.6%)	614 (74.2%)	962 (88.0%)	<0.001	0.731
β	1298 (52.6%)	189 (34.4%)	415 (50.2%)	694 (63.5%)	<0.001	0.989
CCBs	2068 (83.7%)	397 (72.2%)	663 (80.2%)	1008 (92.2%)	<0.001	0.520
Diuretic	1332 (53.9%)	232 (42.2%)	401 (48.5%)	699 (64.0%)	<0.001	0.834

Annotation: BMI, body mass index; SBP, systolic blood pressure; DBP, diastolic blood pressure; DM, diabetes mellitus; ALT, alanine transaminase; AST, aspartate transaminase; Crea, creatinine; BUN, blood urea nitrogen; UA, uric acid; TC, total cholesterol; TG, triglyceride; HDLC, high-density lipoprotein cholesterol; LDLC, low-density lipoprotein cholesterol; ACEI, angiotensin converting enzyme inhibitors; ARB, angiotensin receptor blocker; CCBs, calcium channel blockers; β, β-receptor blocker; IPTW, inverse probability of treatment weighted.

**Table 2:** Association between aspirin groups and different end point events in Cox proportional hazards model.

	Unadjusted Model		Model 1 *		Model 2 ^†^		With IPTW ^‡^	
	HR (95%CI)	*p*	aHR (95%CI)	*p*	aHR (95%CI)	*p*	aHR (95%CI) ^§^	*p ^||^*
MACCE								
Intermittent users	1.40 (0.88–2.22)	0.156	1.17 (0.74–1.86)	0.503	1.13 (0.71–1.80)	0.614	1.21 (0.68–2.14)	0.522
Persistent users	2.79 (1.84–4.22)	<0.001	2.09 (1.37–3.18)	0.001	1.95 (1.26–3.03)	0.003	2.11 (1.23–3.63)	0.007
Ischemic events								
Intermittent users	1.65 (1.00–2.72)	0.052	1.37 (0.83–2.26)	0.224	1.34 (0.80–2.22)	0.266	1.46 (0.78–2.73)	0.239
Persistent users	3.34 (2.12–5.27)	<0.001	2.47 (1.56–3.92)	<0.001	2.38 (1.47–3.84)	<0.001	2.58 (1.42–4.69)	0.002
Cerebrovascular events								
Intermittent users	1.55 (0.94–2.57)	0.088	1.28 (0.77–2.14)	0.335	1.25 (0.75–2.09)	0.398	1.50 (0.82–2.75)	0.193
Persistent users	3.02 (1.91–4.78)	<0.001	2.26 (1.42–3.59)	0.001	2.11 (1.30–3.42)	0.002	2.55 (1.44–4.51)	0.001
Cerebral hemorrhage								
Intermittent users	0.76 (0.20–2.83)	0.682	0.79 (0.21–3.03)	0.736	0.61 (0.15–2.44)	0.484	0.48 (0.10–2.29)	0.359
Persistent users	0.53 (0.14–1.99)	0.351	0.58 (0.15–2.33)	0.446	0.36 (0.09–1.54)	0.170	0.33 (0.07–1.51)	0.152
Cerebral infarction								
Intermittent users	1.81 (1.05–3.14)	0.034	1.48 (0.85–2.57)	0.168	1.46 (0.83–2.54)	0.188	1.83 (0.95–3.52)	0.072
Persistent users	3.58 (2.16–5.93)	<0.001	2.63 (1.58–4.38)	<0.001	2.52 (1.49–4.27)	0.001	3.14 (1.69–5.84)	<0.001
Cardiac events								
Intermittent users	1.21 (0.37–4.03)	0.751	1.15 (0.35–3.85)	0.816	0.99 (0.29–3.34)	0.984	0.64 (0.15–2.80)	0.550
Persistent users	2.80 (0.98–8.04)	0.055	2.38 (0.81–6.94)	0.113	2.04 (0.65–6.34)	0.220	1.43 (0.38–5.45)	0.600
MI								
Intermittent users	1.75 (0.18–16.87)	0.626	1.71 (0.18–16.62)	0.645	1.67 (0.17–16.74)	0.661	1.15 (0.11–12.45)	0.910
Persistent users	3.73 (0.47–29.42)	0.212	3.36 (0.41–27.18)	0.256	3.73 (0.42–33.17)	0.237	2.54 (0.31–20.53)	0.383
Revascularization								
Intermittent users	1.22 (0.31–4.88)	0.778	1.16 (0.29–4.67)	0.834	0.96 (0.24–3.94)	0.959	0.58 (0.10–3.29)	0.536
Persistent users	3.33 (1.00–11.08)	0.050	2.88 (0.85–9.81)	0.090	2.33 (0.64–8.51)	0.201	1.66 (0.35–7.82)	0.523
All-causer death								
Intermittent users	0.59 (0.04–9.44)	0.709	0.42 (0.03–6.95)	0.546	0.53 (0.03–9.26)	0.662	0.40 (0.01–10.73)	0.583
Persistent users	1.56 (0.17–13.99)	0.692	0.83 (0.09–7.88)	0.872	1.50 (0.11–20.32)	0.762	1.02 (0.06–16.17)	0.991
Gastrointestinal bleeding								
Intermittent users	0.27 (0.09–0.77)	0.014	0.24 (0.08–0.70)	0.009	0.25 (0.09–0.74)	0.013	0.31 (0.08–1.16)	0.083
Persistent users	0.63 (0.30–1.35)	0.235	0.53 (0.24–1.16)	0.112	0.57 (0.24–1.34)	0.194	0.66 (0.23–1.86)	0.431

Annotation: MACCE, major adverse cardiovascular and cerebrovascular events; MI, myocardial infarction; IPTW, inverse probability of treatment weighted; HR, hazard ratio; 95%CI, 95% confidence interval; aHR, adjusted hazard ratio. * Model 1 adjusted age, gender, BMI, smoke, baseline SBP. † Model 2 adjusted for variables in model 1 plus ACEI/ARB, β, CCBs, hypoglycemic drugs, lipid-lowering drugs and diuretic. ‡ The model with IPTW adjusted variables are the same as Model 2. § aHR, adjusted HR after IPTW. || *p*, *p*-value after IPTW.

## Data Availability

The data of this study are available from the corresponding author upon reasonable request (including program code for statistical packages).

## Supplementary Materials

The following supporting information can be downloaded at: https://www.mdpi.com/article/10.3390/jcm11237066/s1, Figure S1: Cumulative incidence of aspirin groups in other endpoint events assessed by Kaplan–Meier curves; Figure S2: E-values between continuous aspirin use and different endpoints; Table S1: The description of missing data; Table S2: OSA grade subgroups analysis in different endpoint events; Table S3: Comparison of aspirin use between patients with moderate and severe OSA and patients with mild OSA in different endpoint events; Table S4: Comparison of aspirin use between patients with moderate and severe OSA at different endpoint events; Table S5: Baseline comparison before and after multiple imputation; Table S6: Subgroup stratification and interaction analysis in MACCE; Table S7: Subgroup stratification and interaction analysis in ischemic events; Table S8: Age subgroups analysis in different endpoint events; Table S9: E-values between continuous aspirin use and different endpoint events.
