# Supplementary material for: The Use of Aspirin Increases the Risk of Major Adverse Cardiac and Cerebrovascular Events in Hypertensive Patients with Obstructive Sleep Apnea for the Primary Prevention of Cardiovascular Disease: A Real-World Cohort Study
Source: J Clin Med. 2022 Nov 29;11(23):7066. doi: 10.3390/jcm11237066 (PMC9737230; doi:10.3390/jcm11237066)
Supplement: Supplementary file 1 [file jcm-11-07066-s001.zip › jcm-2004924-supplementary.pdf]

SUPPLEMENTAL MATERIAL

Supplemental Methods

Appendix SA. Definition of aspirin groups

Aspirin groups	Definition
None users	aspirin prescriptions:<br>in inpatient orders = 0, outpatient clinics records = 0, pharmacy records = 0
Intermittent users	aspirin prescription:<br>1) long-term inpatient doctor's order = 1 time/year + 0 times ≤ outpatient or pharmacy prescription ≤ 2 times/year, or<br>2) long-term inpatient doctor's order = 0 times/year + 1 ≤ outpatient or pharmacy prescription ≤ 3 times/year
Persistent users	aspirin prescription:<br>1) inpatient long-term medical orders ≥ 2 times/year + outpatient or pharmacy prescriptions ≥ 2 times/year, or<br>2) inpatient long-term medical orders = 1 time/year + outpatient or pharmacy prescriptions ≥ 3 times/year, or<br>3) inpatient long-term medical orders = 0 times/year + pharmacy prescription ≥ 4 times/year

Appendix SB. The 10th edition of the International Classification of Disease (ICD-10) codes or International Classification of Diseases Clinical Modification of 9th Revision Operations and Procedures (ICD-9-CM-3) codes for disease diagnosis from hospital discharge and mortality records

Disease type	ICD-10 codes
Hypertension	I10-I15
Obstructive sleep apnea	I27.9
Ischemic heart disease	I20-I25
Heart failure	I50
Stroke	I61-I63
Gastrointestinal bleeding	K92.0-K92.2
Cerebrovascular illness	I60-I66, I69
Diabetes	E10-11
Unspecified kidney failure	N19
Chronic kidney disease	N18
Chronic obstructive pulmonary disease	J44.9, J44.151
Respiratory failure	J96.901, J96.151, J96.051, G93.818
Liver failure	K72.001-002, K72.901-903, K72.151, K72.051
Disease type	ICD-9-CM-3 codes
Cardiac revascularization	36.0-36.1, 00.45-00.48, 00.66, 17.55

Supplementary Table S1. The description of missing data

Variables	Non-missing	Missing
Sex	2470	0
Age	2470	0
Duration of hypertension	2470	0
Smoke	2470	0
Drink	2470	0
Baseline DBP	2462	8
Baseline SBP	2466	4
BMI	2057	413
Waist	2024	446
OSA grade	2470	0
Baseline DM	2470	0
ALT	2076	394
AST	2076	394
Crea	2366	104
BUN	2365	105
UA	1989	481
TC	1929	541
TG	1931	539
HDLC	1929	541
LDLC	1929	541
Hypoglycemic drugs	2470	0
Lipidlowering drugs	2470	0
ACEI/ARB	2470	0
β	2470	0
CCBs	2470	0
Anti-platelet	2470	0
Diuretic	2470	0

Annotation: BMI, body mass index; SBP, systolic blood pressure; DBP, diastolic blood pressure; DM, diabetes mellitus; ALT, alanine transaminase; AST, aspartate transaminase; Crea, creatinine; BUN, blood urea Nitrogen; UA, uric acid; TC, total cholesterol; TG, triglyceride; HDLC, High density liptein cholesterol; LDLC, low density lipoprotein cholesterol; ACEI, angiotensin converting enzyme inhibitors; ARB, angiotensin receptor blocker; CCBs, calcium channel blockers; β ,β-receptor blocker;

Supplementary Table S2. OSA grade subgroups analysis in different endpoint events

	None users	Intermittent users	*p* for interaction	Persistent users	*p* for interaction
MACCE			0.527		0.052
Mild	Ref	2.79(0.99-7.84)		5.86(2.15-15.95)	
Moderate	Ref	0.48(0.16-1.46)		1.09(0.41-2.87)	
Severe	Ref	1.42(0.57-3.52)		1.71(0.73-4.01)	
Ischemic events			0.682		0.026
Mild	Ref	2.98(0.99-8.93)		6.42(2.22-18.61)	
Moderate	Ref	0.79(0.22-2.79)		1.69(0.53-5.37)	
Severe	Ref	1.41(0.55-3.61)		1.70(0.71-4.08)	
Cerebrovascular events			0.590		0.047
Mild	Ref	4.00(1.25-12.81)		7.61(2.40-24.10)	
Moderate	Ref	0.75(0.25-2.20)		1.52(0.59-3.94)	
Severe	Ref	1.39(0.51-3.84)		1.78(0.70-4.54)	
Cerebral infarction			0.675		0.033
Mild	Ref	4.48(1.25-16.11)		8.76(2.47-31.10)	
Moderate	Ref	1.27(0.39-4.08)		2.43(0.84-7.05)	
Severe	Ref	1.37(0.47-3.97)		1.75(0.66-4.67)	
Gastrointestinal bleeding			0.639		0.250
Mild	Ref	0.11(0.01-1.81)		0.56(0.14-2.19)	
Moderate	Ref	2.07(0.24-17.94)		2.26(0.30-17.29)	
Severe	Ref	0.11(0.01-1.26)		0.28(0.05-1.63)	

Annotation: OSA, obstructive sleep apnea; MACCE, Major adverse cardiovascular and cerebrovascular events.

Supplementary Table S3. Comparison of aspirin use between patients with moderate&severe OSA and patients with mild OSA in different endpoint events

	None users	Intermittent users	*p* for interaction	Persistent users	*p* for interaction
MACCE			0.061		0.016
Mild	Ref	2.79(0.99-7.84)		5.86(2.15-15.95)	
Moderate&Severe	Ref	0.86(0.43-1.71)		1.37(0.73-2.59)	
Ischemic events			0.125		0.039
Mild	Ref	2.98(0.99-8.93)		6.42(2.22-18.61)	
Moderate&Severe	Ref	1.08(0.50-2.32)		1.72(0.84-3.52)	
Cerebrovascular events			0.053		0.023
Mild	Ref	4.00(1.25-12.81)		7.61(2.40-24.10)	
Moderate&Severe	Ref	1.04(0.50-2.14)		1.68(0.87-3.23)	
Cerebral infarction			0.101		0.046
Mild	Ref	4.48(1.25-16.11)		8.76(2.47-31.10)	
Moderate&Severe	Ref	1.30(0.59-2.84)		2.08(1.02-4.25)	
Gastrointestinal bleeding			0.560		0.692
Mild	Ref	0.11(0.01-1.81)		0.56(0.14-2.19)	
Moderate&Severe	Ref	0.44(0.09-2.30)		0.67(0.17-2.63)	

Annotation: OSA, obstructive sleep apnea; MACCE, Major adverse cardiovascular and cerebrovascular events.

Supplementary Table S4. Comparison of aspirin use between patients with moderate and severe OSA at different endpoint events

	None users	Intermittent users	*p* for interaction	Persistent users	*p* for interaction
MACCE			0.120		0.460
Moderate	Ref	0.48(0.16-1.46)		1.09(0.41-2.87)	
Severe	Ref	1.42(0.57-3.52)		1.71(0.73-4.01)	
Ischemic events			0.415		0.922
Moderate	Ref	0.79(0.22-2.79)		1.69(0.53-5.37)	
Severe	Ref	1.41(0.55-3.61)		1.70(0.71-4.08)	
Cerebrovascular events			0.361		0.737
Moderate	Ref	0.75(0.25-2.20)		1.52(0.59-3.94)	
Severe	Ref	1.39(0.51-3.84)		1.78(0.70-4.54)	
Cerebral infarction			0.849		0.740
Moderate	Ref	1.27(0.39-4.08)		2.43(0.84-7.05)	
Severe	Ref	1.37(0.47-3.97)		1.75(0.66-4.67)	
Gastrointestinal bleeding			0.072		0.107
Moderate	Ref	2.07(0.24-17.94)		2.26(0.30-17.29)	
Severe	Ref	0.11(0.01-1.26)		0.28(0.05-1.63)	

Annotation: OSA, obstructive sleep apnea; MACCE, Major adverse cardiovascular and cerebrovascular events.

Supplementary Table S5. Baseline comparison before and after multiple imputation

	before multiple imputation	after multiple imputation	*p*
	n=2470	*n*=2470	
Gender, *n(%)*			1.000
Female	558 (22.6%)	558 (22.6%)	
Male	1912 (77.4%)	1912 (77.4%)	
Age, y *M[P25, P75]*	48.0 [42.0;55.0]	48.0 [42.0;55.0]	1.000
Duration of hypertension,y *M[P25, P75]*	109 [74;154]	109 [74;154]	1.000
Smoke (ever), *n(%)*	1209 (48.9%)	1209 (48.9%)	1.000
Drink (ever), *n(%)*	1108 (44.9%)	1108 (44.9%)	1.000
Baseline DBP, mmHg *M[P25, P75]*	92[83;102.]	92 [83;102]	0.987
Baseline SBP, mmHg *M[P25, P75]*	146 [134;162]	146 [134;162]	0.991
BMI, kg/㎡ *mean[SD]*	28.4 [26.1;30.9]	28.4 [26.1;30.8]	0.677
BMI groups, *n(%)*			0.886
＜28kg/㎡	1108 (44.9%)	1114 (45.1%)	
≥28kg/㎡	1362 (55.1%)	1356 (54.9%)	
Waist, cm *M[P25, P75]*	102.0 [95.0;109.0]	101.0 [95.0;109.0]	0.818
OSA grade, *n(%)*			1.000
Mild	862 (34.9%)	862 (34.9%)	
Moderate	764 (30.9%)	764 (30.9%)	
Severe	844 (34.2%)	844 (34.2%)	
ALT, U/L *M[P25, P75]*	26.0 [18.0;39.0]	26.0 [18.0;39.0]	0.829
AST, U/L *M[P25, P75]*	19.0 [16.0;24.7]	19.0 [16.0;25.0]	0.697
Crea, µmol/L *M[P25, P75]*	68.1 [58.7;78.5]	68.1 [58.7;78.4]	0.961
BUN, mmol/L *M[P25, P75]*	5.0 [4.2;5.9]	5.0 [4.2;5.9]	0.868
UA, µmol/L *M[P25, P75]*	369 [309;429]	369 [309;431]	0.767
TC, mmol/L *M[P25, P75]*	4.5 [3.9;5.1]	4.5 [3.9;5.1]	0.588
TG, mmol/L *M[P25, P75]*	1.8 [1.3;2.6]	1.8 [1.3;2.6]	0.268
HDLC, mmol/L	0.9 [0.8;1.1]	0.9 [0.8;1.1]	0.757

M[P25, P75]			
LDLC, mmol/L *M[P25, P75]*	2.7 [2.2;3.3]	2.7 [2.2;3.3]	0.774

Annotation: BMI, body mass index; SBP, systolic blood pressure; DBP, diastolic blood pressure; ALT, alanine transaminase; AST, aspartate transaminase; Crea, creatinine; BUN, blood urea Nitrogen; UA, uric acid; TC, total cholesterol; TG, triglyceride; HDLC, High density liptein cholesterol; LDLC, low density lipoprotein cholesterol.

Supplementary Table S6. Subgroup stratification and interaction analysis in MACCE

	n	None users	Intermittent users	*p* for interaction	Persistent users	*p* for interaction
Gender				0.330		0.074
Female	558	Ref	3.85(0.47-31.70)		13.10(1.65-104.0)	
Male	1912	Ref	1.18(0.64-2.17)		1.72(0.97-3.07)	
Age				0.054		0.001
<50	1407	Ref	0.98(0.43-2.24)		1.37(0.64-2.95)	
≥50	1063	Ref	1.32(0.61-2.84)		2.69(1.34-5.39)	
BMI				0.118		0.491
<28kg/㎡	1114	Ref	1.83(0.85-3.96)		2.66(1.28-5.51)	
≥28kg/㎡	1356	Ref	0.62(0.28-1.36)		1.57(0.77-3.19)	
Duration of hypertension groups				0.053		0.303
≤4 years	735	Ref	0.66(0.23-1.87)		1.74(0.70-4.32)	
＞4 years,≤8 years	994	Ref	1.99(0.88-4.51)		2.40(1.09-5.26)	
＞8 years	741	Ref	0.98(0.35-2.75)		2.34(0.85-6.42)	
Baseline DM				0.738		0.802
No	1831	Ref	1.14(0.57-2.26)		2.03(1.06-3.90)	
Have	639	Ref	1.35(0.49-3.76)		2.28(0.90-5.75)	
Smoke				0.482		0.271
Never	1261	Ref	1.54(0.62-3.86)		3.21(1.35-7.62)	
Ever	1209	Ref	1.00(0.48-2.10)		1.66(0.84-3.26)	
Drink				0.647		0.412
Never	1362	Ref	1.36(0.59-3.15)		2.64(1.16-6.04)	
Ever	1108	Ref	1.05(0.50-2.19)		1.70(0.85-3.41)	
UA				0.579		0.501
≤420μmol/L	1755	Ref	1.36(0.71-2.59)		2.44(1.33-4.47)	
>420μmol/L	715	Ref	0.93(0.31-2.73)		1.59(0.59-4.26)	
Lipidlowering drugs				0.112		0.457
No	429	Ref	2.86(0.86,9.54)		2.48(0.62-9.99)	
Have	2041	Ref	1.09(0.59-2.02)		2.01(1.13-3.57)	
Hypoglycemic drugs				0.208		0.153
No	1009	Ref	1.93(0.76-4.95)		3.58(1.50-8.52)	
Have	1461	Ref	0.92(0.45-1.89)		1.52(0.78-2.94)	
ACEI/ARB				0.818		0.865
No	544	Ref	1.53(0.49-4.80)		2.27(0.74-6.98)	
Have	1926	Ref	1.15(0.60-2.21)		2.02(1.10-3.71)	
β				0.236		0.184
No	1172	Ref	1.91(0.86-4.23)		3.48(1.64-7.36)	
Have	1298	Ref	0.93(0.44-1.95)		1.55(0.77-3.12)	

CCB				0.485		0.740
No	402	Ref	1.92(0.63-5.89)		1.85(0.56-6.07)	
Have	2068	Ref	1.10(0.58-2.08)		2.13(1.17-3.88)	
Diuretic				0.959		0.842
No	1138	Ref	1.29(0.54-3.09)		2.35(1.03-5.36)	
Have	1332	Ref	1.15(0.54-2.45)		1.94(0.96-3.92)	

Annotation: BMI, body mass index; DM, diabetes mellitus; UA, uric acid; ACEI, angiotensin converting enzyme inhibitors; ARB, angiotensin receptor blocker; CCB, calcium channel blockers; β, β-receptor blocker; MACCE, Major adverse cardiovascular and cerebrovascular events.

Supplementary Table S7. Subgroup stratification and interaction analysis in Ischemic events

	n	None users	Intermittent users	*p* for interaction	Persistent users	*p* for interaction
Age				0.055		0.003
<50	1407	Ref	1.06(0.42-2.65)		1.58(0.67-3.71)	
≥50	1063	Ref	1.72(0.75-3.95)		3.39(1.58-7.28)	
BMI				0.378		0.997
<28kg/㎡	1114	Ref	1.80(0.83-3.89)		2.61(1.26-5.41)	
≥28kg/㎡	1356	Ref	0.84(0.34-2.10)		2.19(0.94-5.12)	
Duration of hypertension groups				0.151		0.107
≤4 years	735	Ref	1.20(0.36-3.97)		3.19(1.05-9.73)	
＞4 years,≤8 years	994	Ref	2.43(0.99-5.92)		2.93(1.24-6.90)	
＞8 years	741	Ref	0.98(0.35-2.76)		2.31(0.84-6.35)	
Baseline DM				0.798		0.990
No	1831	Ref	1.39(0.65-2.96)		2.60(1.26-5.37)	
Have	639	Ref	1.62(0.55-4.80)		2.62(0.96-7.13)	
Smoke				0.291		0.140
Never	1261	Ref	2.37(0.77-7.28)		5.15(1.76-15.07)	
Ever	1209	Ref	1.12(0.52-2.44)		1.84(0.90-3.76)	
Drink				0.988		0.727
Never	1362	Ref	1.44(0.60-3.45)		2.86(1.20-6.82)	
Ever	1108	Ref	1.43(0.70-3.27)		2.36(1.07-5.17)	
UA				0.427		0.311
≤420μmol/L	1755	Ref	1.80(0.89-3.63)		3.33(1.71-6.48)	
>420μmol/L	715	Ref	1.02(0.328-3.17)		1.67(0.59-4.76)	
Lipidlowering drugs				0.135		0.461
No	429	Ref	3.54(0.98-12.80)		3.24(0.75-14.0)	
Have	2041	Ref	1.31(0.67-2.57)		2.45(1.30-4.63)	
Hypoglycemic drugs				0.133		0.114
No	1009	Ref	2.78(1.02-7.52)		4.96(1.94-12.72)	
Have	1461	Ref	1.06(0.49-2.32)		1.80(0.87-3.70)	
ACEI/ARB				0.991		0.950
No	544	Ref	1.66(0.50-5.53)		2.45(0.75-8.04)	
Have	1926	Ref	1.43(0.70-2.93)		2.54(1.28-5.02)	
β				0.372		0.366
No	1172	Ref	2.12(0.92-4.88)		3.80(1.72-8.43)	

Have	1298	Ref	1.16(0.51-2.65)		2.00(0.89-4.46)	
CCB				0.612		0.673
No	402	Ref	2.10(0.64-6.93)		2.11(0.61-7.35)	
Have	2068	Ref	1.33(0.66-2.70)		2.64(1.35-5.15)	
Diuretic				0.521		0.672
No	1138	Ref	1.28(0.52-3.12)		2.44(1.05-5.69)	
Have	1332	Ref	1.76(0.72-4.32)		2.93(1.25-6.86)	

Annotation: BMI, body mass index; DM, diabetes mellitus; UA, uric acid; ACEI, angiotensin converting enzyme inhibitors; ARB, angiotensin receptor blocker; CCB, calcium channel blockers; β, β-receptor blocker; MACCE, Major adverse cardiovascular and cerebrovascular events.

Supplementary Table S8. Age subgroups analysis in different endpoint events

	None users	Intermittent users	*p* for interaction	Persistent users	*p* for interaction
MACCE			0.054		0.001
Age<50	Ref	0.98(0.43-2.24)		1.37(0.64-2.95)	
Age≥50	Ref	1.32(0.61-2.84)		2.69(1.34-5.39)	
Ischemic events			0.055		0.003
Age<50	Ref	1.06(0.42-2.65)		1.58(0.67-3.71)	
Age≥50	Ref	1.72(0.75-3.95)		3.39(1.58-7.28)	
Cerebrovascular events			0.195		0.012
Age<50	Ref	1.88(0.85-4.20)		2.49(1.15-5.41)	
Age≥50	Ref	1.30(0.58-2.91)		2.50(1.20-5.23)	
Cerebral hemorrhage			0.282		0.958
Age<50	Ref	2.35(0.39-14.29)		0.74(0.09-5.83)	
Age≥50	Ref	0.16(0.02-1.22)		0.24(0.05-1.22)	
Cerebral infarction			0.214		0.031
Age<50	Ref	1.96(0.82-4.71)		2.77(1.19-6.42)	
Age≥50	Ref	1.73(0.71-4.18)		3.22(1.41-7.35)	
Cardiac events			0.110		0.029
Age<50	Ref	0.34(0.06-1.92)		0.50(0.11-2.34)	
Age≥50	Ref	1.35(0.10-17.30)		4.96(0.47-52.35)	
Gastrointestinal bleeding			0.728		0.789
Age<50	Ref	0.36(0.09-1.50)		0.74(0.23-2.41)	
Age≥50	Ref	0.27(0.04-2.01)		0.69(0.14-3.26)	

Annotation: MACCE, Major adverse cardiovascular and cerebrovascular events.

Supplementary Table S9 E-values between continuous aspirin use and different endpoint events

	MACCE	Ischemic events	Cerebrovascular events	Cerebral infarction
aHR*(95%CI)	2.11 (1.23-3.63)	2.58 (1.42-4.69)	2.55 (1.44-4.51)	3.14 (1.69-5.84)
E-value for HR estimate	3.64	4.60	4.54	5.73
E-value for upper limit of 95% CI	1.76	2.19	2.24	2.77

Annotation: MACCE, Major adverse cardiovascular and cerebrovascular events; HR, hazard ratio; 95%CI, 95% confidence interval.
aHR*: adjusted *HR* after inverse probability of treatment weighted.

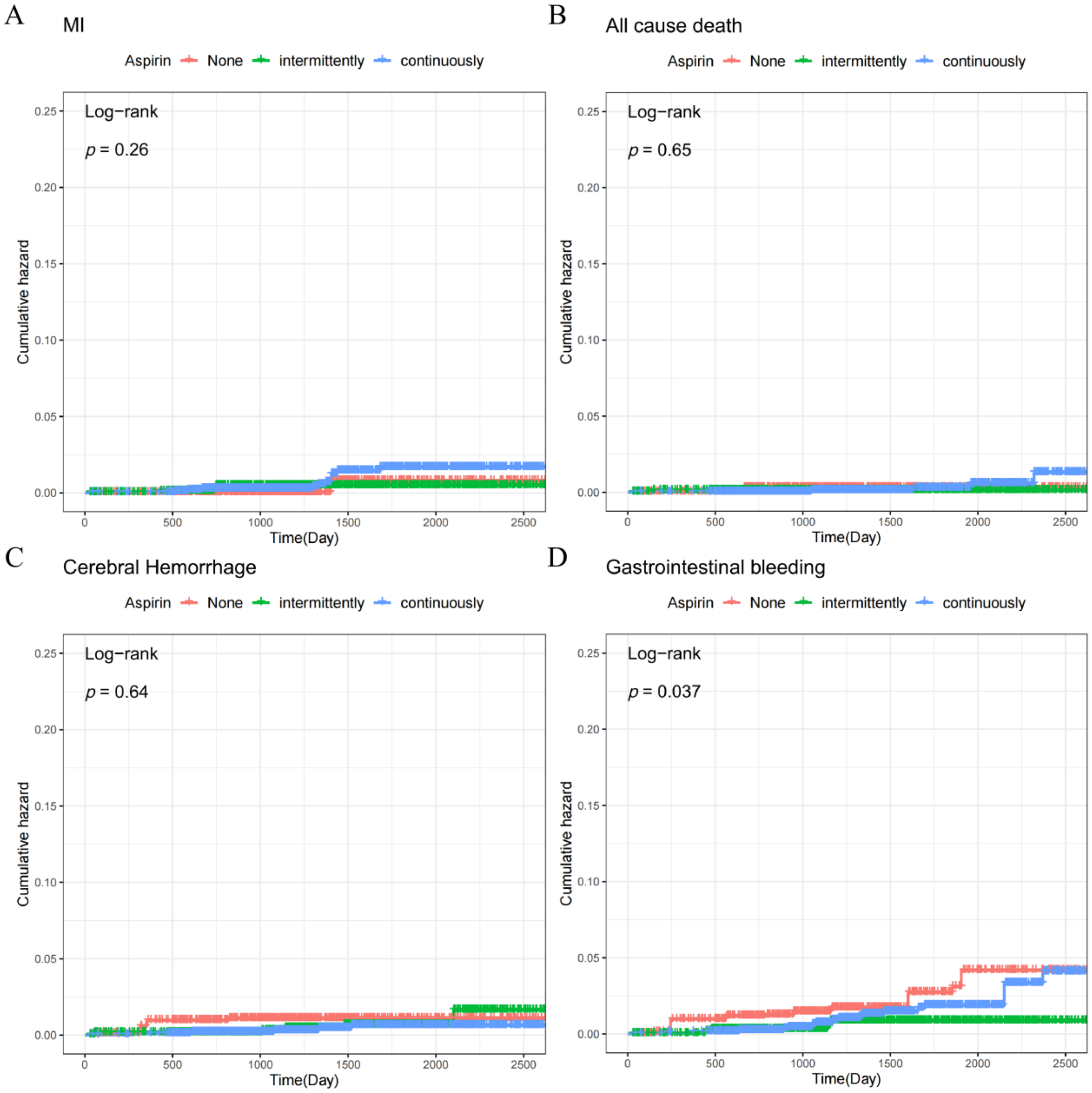

Figure S1. Cumulative incidence of aspirin groups in other endpoint events assessed by Kaplan & Meier curves

Annotation: MI, myocardial infarction; (A) MI; (B) All cause death; (C) Cerebral Hemorrhage; (D) Gastrointestinal bleeding.

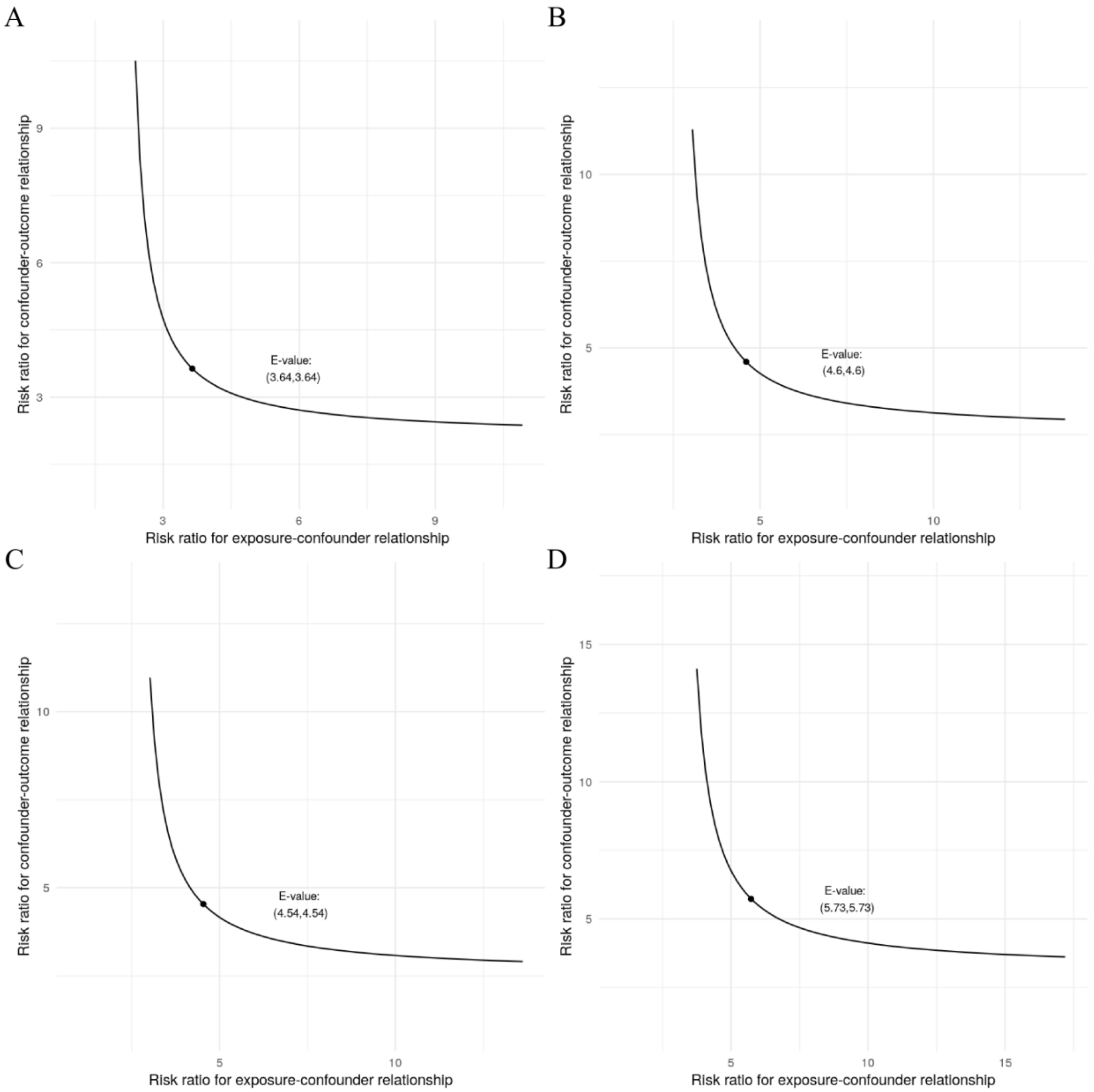

Figure S2. E-values between continuous aspirin use and different endpoints

Annotation: MACCE, Major adverse cardiovascular and cerebrovascular events. (A) MACCE; (B) Ischemic Events; (C) Cerebrovascular Events; (D) Cerebral Infarction.
